# Editorial: Endothelial dysfunction in metabolic disorders: mechanisms, biomarkers, and emerging therapeutics

**DOI:** 10.3389/fphar.2026.1863717

**Published:** 2026-05-07

**Authors:** Rajashekhar Gangaraju

**Affiliations:** 1 Department of Ophthalmology, University of Tennessee Health Science Center, Memphis, TN, United States; 2 Department of Anatomy and Neurobiology, University of Tennessee Health Science Center, Memphis, TN, United States

**Keywords:** diabetes, endothelial dysfunction, eNOS (endothelial nitric oxide synthase), methyglyoxal, O-GlcNAc glycosylation

Endothelial dysfunction is increasingly recognized as a central mechanism through which diverse metabolic insults contribute to both microvascular and macrovascular injury (Yuchen et al., 2023; Lenin et al., 2018). The Research Topic, “Endothelial Dysfunction in Metabolic Disorders: Mechanisms, Biomarkers, and Emerging Therapeutics,” was curated to elucidate underlying mechanisms, refine biomarker identification, and highlight emerging therapeutic strategies across organ systems. The three primary objectives were: (1) to dissect cellular and molecular pathways by which metabolic stressors disrupt endothelial homeostasis; (2) to identify circulating, imaging, and functional biomarkers that detect early endothelial injury and stratify cardiometabolic risk; and (3) to showcase novel pharmacological and non-pharmacological interventions that restore or preserve endothelial function.

By integrating mechanistic reviews, clinical translational studies, and preclinical research, this Research Topic bridges traditional cardiovascular paradigms with renal, diabetic, and nutritional-metabolic perspectives, emphasizing the bidirectional relationship between metabolic dysregulation and vascular health. Tang et al. (PMID: 41716809) provide a comprehensive synthesis of the role of endothelial cells in regulating renal microcirculation and the consequences of their dysfunction in kidney diseases. Their review details how endothelial cells regulate microvascular tone, perfusion balance, and barrier properties, and explains how injury impairs relaxation, induces microvascular rarefaction, and promotes renal pathology. Importantly, the review demonstrates that microcirculatory disorders in the kidney often originate from primary endothelial dysfunction, rather than being solely downstream effects of glomerular injury, thereby positioning the renal endothelium as both a sensor and effector of metabolic and hemodynamic stress.

Building on these systemic insights, Li et al. (PMID: 41476791) examine the role of O-GlcNAcylation, a specific post-translational modification of endothelial nitric oxide synthase (eNOS), in a high-salt diet model of thoracic aortic endothelial dysfunction in mice. Their findings indicate that high salt intake impairs endothelial function and elevates diastolic blood pressure, coinciding with increased O-GlcNAc modification of eNOS. These results identify nutrient- and salt-sensitive glycosylation pathways as critical modulators of nitric oxide bioavailability. This study situates salt-induced hypertension within a broader metabolic framework, demonstrating how excessive dietary sodium interacts with glucose- and hexosamine-dependent signaling to induce endothelial dysfunction.

Together, these two studies underscore that endothelial dysfunction in metabolic disorders is highly context- and vascular-bed specific. Renal microvessels and large conduit arteries exhibit distinct vulnerabilities and adaptive responses, yet share common mechanisms such as impaired nitric oxide signaling, oxidative stress, and microvascular remodeling (Rajashekhar et al., 2007; Arendshorst et al., 2024; Shaito et al., 2022; Wang and He, 2020; Rocha and Adams, 2009).

Li et al. (PMID: 41480358) further explore the renal theme by analyzing the specificity of endothelial cells in the context of endothelial dysfunction in diabetic kidney disease (DKD) and their interactions with neighboring cells. This review underscores that a comprehensive understanding of DKD requires consideration of the heterogeneity among endothelial cell subtypes across glomerular, peritubular, and larger renal vessels, each exhibiting unique transcriptional, metabolic, and functional profiles in response to chronic hyperglycemia and insulin resistance. The review highlights the reciprocal communication among endothelial cells, podocytes, mesangial cells, and tubular epithelium, mediated by cytokines, growth factors, extracellular vesicles, and metabolic signals. This interplay amplifies endothelial injury and accelerates DKD progression. By emphasizing cell–cell crosstalk, the work shifts the perspective from a vessel-centric model of diabetic microangiopathy to a multicellular network framework, informing the development of therapies that target endothelial dysfunction while also modulating responses in neighboring cells.

Yu et al. (PMID: 41716315) address a central objective of this Research Topic by investigating clinically actionable biomarkers of endothelial dysfunction in metabolic disease. In a cross-sectional cohort of 250 patients with type 2 diabetes, participants were stratified by endothelial function status using flow-mediated dilation (FMD), a widely accepted non-invasive marker of endothelial health. The study demonstrates that serum methylglyoxal (MGO), a reactive dicarbonyl elevated in hyperglycemic states, is significantly higher in patients with endothelial dysfunction and exhibits a strong negative correlation with FMD (Spearman R = −0.611, p < 0.001), independent of age and sex. Receiver operating characteristic analysis further supports MGO as a predictive biomarker of impaired endothelial function in type 2 diabetes. These findings connect a metabolic byproduct of chronic hyperglycemia to a functional vascular outcome, suggesting that incorporating MGO into risk stratification may enhance identification of diabetic patients at highest vascular risk and inform targeted endothelial-protective interventions.

Several converging therapeutic themes are evident across these contributions. Reviews on renal microcirculation and DKD highlight opportunities for interventions that preserve endothelial integrity, normalize microvascular perfusion, and disrupt pathological crosstalk with neighboring cells, potentially mitigating the progression of kidney disease in metabolic contexts. The finding that high-salt intake promotes O-GlcNAcylation of eNOS suggests that therapies targeting nutrient-sensitive glycosylation pathways or enhancing nitric oxide bioavailability may be especially effective in salt-sensitive hypertension and cardiometabolic disease. At the biomarker level, MGO is now part of an expanding panel of circulating and functional indices that detect early endothelial injury prior to overt cardiovascular events. This aligns with ongoing efforts to integrate metabolic, inflammatory, and vascular biomarkers into precision cardiometabolic medicine. In light of recent research identifying endothelial dysfunction as both a hallmark and driver of cardiometabolic disease, including heart failure with preserved ejection fraction and atherosclerotic cardiovascular disease (Cornuault et al., 2022; Xu et al., 2021; Gorica et al., 2025), this Research Topic reinforces the view that restoring endothelial health constitutes both a vascular and systemic metabolic intervention.

Collectively, the articles in this Research Topic provide a nuanced and integrated perspective of endothelial dysfunction as a dynamic, organ-specific, and metabolically regulated process that is both measurable and modifiable, and central to the prevention and treatment of cardiometabolic disease. The future integration of advanced platforms, such as exosomes as biomarkers or delivery vehicles (Chen et al., 2024), organoids, and iPSC-derived endothelial cells, together with high-resolution techniques like RNA sequencing and spatial transcriptomics, will substantially enhance mechanistic understanding. These technologies are anticipated to reveal previously unrecognized endothelial subpopulations and microenvironmental niches, ultimately enabling more precise, tissue-specific, and stage-specific therapeutic strategies for metabolic vascular disease.
